# Evaluation of the Accuracy of Smartphone Medical Calculation Apps

**DOI:** 10.2196/jmir.3062

**Published:** 2014-02-03

**Authors:** Rachel Bierbrier, Vivian Lo, Robert C Wu

**Affiliations:** ^1^University Health NetworkCentre for Innovation in Complex CareToronto, ONCanada; ^2^University Health NetworkDepartment of General Internal MedicineToronto, ONCanada

**Keywords:** cellular phone, mobile phone, mhealth, medical informatics applications, software, computers, handheld

## Abstract

**Background:**

Mobile phones with operating systems and capable of running applications (smartphones) are increasingly being used in clinical settings. Medical calculating applications are popular mhealth apps for smartphones. These include, for example, apps that calculate the severity or likelihood of disease-based clinical scoring systems, such as determining the severity of liver disease, the likelihood of having a pulmonary embolism, and risk stratification in acute coronary syndrome. However, the accuracy of these apps has not been assessed.

**Objective:**

The objective of this study was to evaluate the accuracy of smartphone-based medical calculation apps.

**Methods:**

A broad search on Google Play, BlackBerry World, and the iTunes App Store was conducted to find medical calculation apps for smartphones. The list of apps was narrowed down based on inclusion and exclusion criteria focusing on functions thought to be relevant by a panel of general internists (number of functions =13). Ten case values were inputted for each function and were compared to manual calculations. For each case, the correct answer was assigned a score of 1. A score for the 10 cases was calculated based on the accuracy of the results for each function on each app.

**Results:**

We tested 14 apps and 13 functions for each app if that function was available. We conducted 10 cases for each function for a total of 1240 tests. Most functions tested on the apps were accurate in their results with an overall accuracy of 98.6% (17 errors in 1240 tests). In all, 6 of 14 (43%) apps had 100% accuracy. Although 11 of 13 (85%) functions had perfect accuracy, there were issues with 2 functions: the Child-Pugh scores and Model for End-Stage Liver Disease (MELD) scores on 8 apps. Approximately half of the errors were clinically significant resulting in a significant change in prognosis (8/17, 47%).

**Conclusions:**

The results suggest that most medical calculating apps provide accurate and reliable results. The free apps that were 100% accurate and contained the most functions desired by internists were CliniCalc, Calculate by QxMD, and Medscape. When using medical calculating apps, the answers will likely be accurate; however, it is important to be careful when calculating MELD scores or Child-Pugh scores on some apps. Despite the few errors found, greater scrutiny is warranted to ensure full accuracy of smartphone medical calculator apps.

## Introduction

Smartphones are rapidly being adopted into the medical field. A recent survey found that 79% of medical students and 75% of postgraduate trainees owned smartphones [1]. One important use of smartphones is to aid in diagnosis, prognosis, and treatment of medical conditions. Apps can aid in diagnosis by providing a reference to staging systems, such as the severity staging of chronic obstructive pulmonary disease (COPD), or can provide rapid access to published algorithms in decision making. These reference or decision support functions that perform minimal calculations are typically considered to be at low risk of causing errors [2].

There are increasing numbers of clinical scoring systems that can include calculations, such as determining the severity of liver disease (Model for End-Stage Liver Disease, MELD), the likelihood of having a pulmonary embolism (Wells’ Score for Pulmonary Embolism), and risk stratification in acute coronary syndrome (the thrombolysis in myocardial infarction, TIMI, score for non-ST elevation myocardial infarction, NSTEMI) [3-5]. Smartphone applications can make calculating these scores easier by providing information rapidly after performing a calculation using patient-specific data. Indeed, medical calculation apps are one of the most-used apps by doctors, often used several times per day [1]. Medical calculation apps can be considered to be of higher complexity because they do not just present previously published information, but may perform complex calculations based on user input. This increases the risk of error.

Health care professionals rely on decision-making aids such as medical apps, yet their accuracy has not been verified. The American Food and Drug Administration (FDA) has attempted to eliminate the distribution of faulty apps related to health care [6]. It is critical that apps used in clinical settings are accurate because the scoring results can impact a clinician’s decision. Unfortunately, there is limited literature on the accuracy of smartphone medical calculators with the current evidence being highly specialized [7,8]. The purpose of our study was to assess the accuracy of general medical calculating apps on smartphones.

## Methods

### Definitions and Search Strategy

For the purpose of the study, an *app* was defined as a smartphone medical app. A *function* was defined as calculation that can be conducted on the app by inputting clinical data or observation. *General internists* were defined as specialists who apply scientific knowledge and clinical expertise to the diagnosis, treatment, and compassionate care of adults across the spectrum from health to complex illness [9].

**Figure 1:**
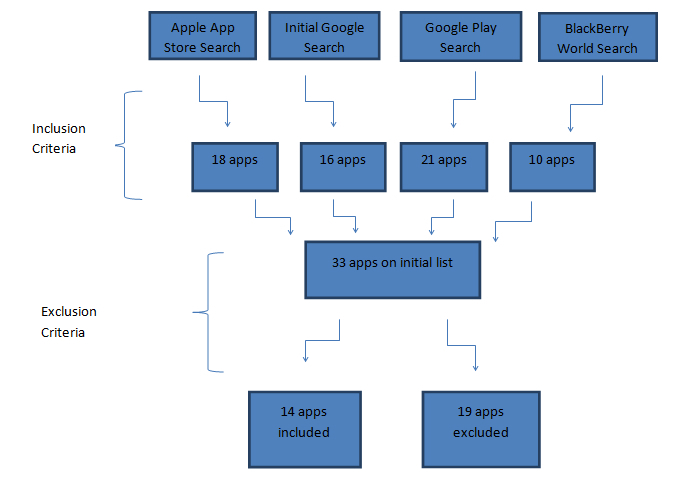
Breakdown of process to select apps for testing.

Online searches were performed to acquire apps relevant to the study. The Google search contained the following keywords: “medical calculator apps,” “apps medical calculator,” “smartphone medical apps,” “medical + smartphone + apps,” and “medical + smartphone.” The first 5 pages of each of the searches were examined, with each page containing 10 links to websites. The keywords “medical” and “medical calculators” were then entered into the search fields of Google Play, BlackBerry World, and the iTunes App Store. The first 10 pages of each search on Google Play (24 apps per page) and BlackBerry World (6 apps per page) were examined. The first 30 rows (8 apps per row) of each search keyword were examined in the App Store for both iPhone and iPad. A complete breakdown of the app search can be found in Figure 1.

### Inclusion and Exclusion Criteria

Two rounds of selection of apps occurred to acquire the final list of apps used for testing. The first round occurred as the apps were reviewed in the initial Google and App Store searches. Apps were included if they met the inclusion criteria of the study. Apps had to have a medical calculating smartphone app with 3 or more calculating functions.

Exclusion criteria were applied once the first draft of apps was compiled. This method of narrowing down the apps dealt with specific calculation functions of the apps focusing on apps in which a general internist would be interested. From all the smartphone apps from round 1, we compiled a list of all calculation functions. We provided this list of all calculation functions to 5 internists and asked them which functions they would want on a medical calculation application. Apps were excluded if it did not contain at least half of the functions selected by 5 physicians.

### Testing

**Figure 2:**
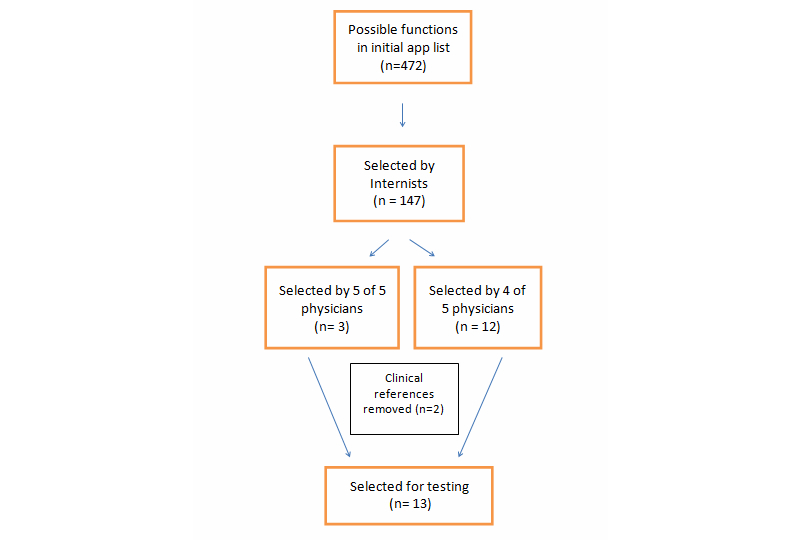
Breakdown of process to select functions for testing.

The medical apps were all downloaded in July 2013. To determine the functions to test, we used the preferred list of functions selected by the general internists. For functions to be tested, it had to be selected by at least 4 of the 5 internists. The selection process of functions is shown in Figure 2. Out of 476 calculating functions that were found on the apps, 147 (30.9%) were selected by 5 internists as useful functions they would want to have on an app. This list was then narrowed down further based on the degree of overlap to 15 functions (Figure 2). The Canadian Cardiovascular Society (CCS) Angina Score and the GOLD Classification of COPD were removed from the list of functions to test because they were classification systems without any calculations. A list of all calculation functions and descriptions is shown in Table 1.

**Figure 3:**
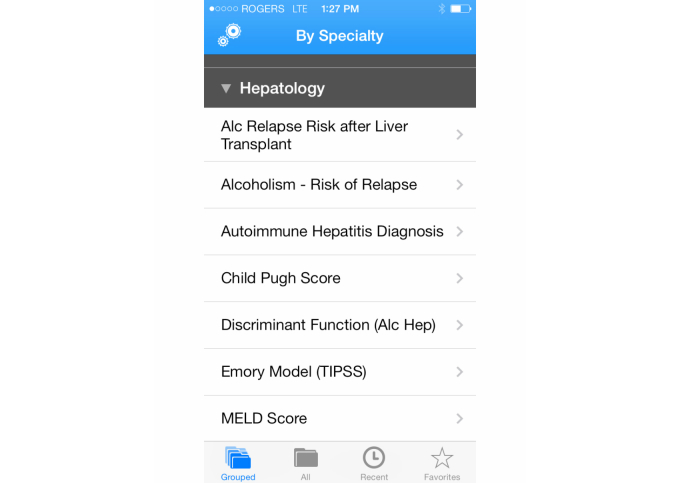
Calculations available on the Calculate by QxMD app.

**Figure 4:**
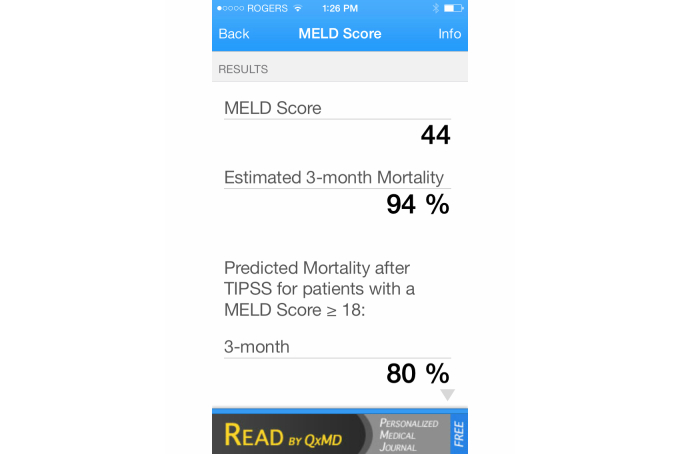
Example of a Model for End-Stage Liver Disease (MELD) Score calculation on the Calculate by QxMD app.

**Figure 5:**
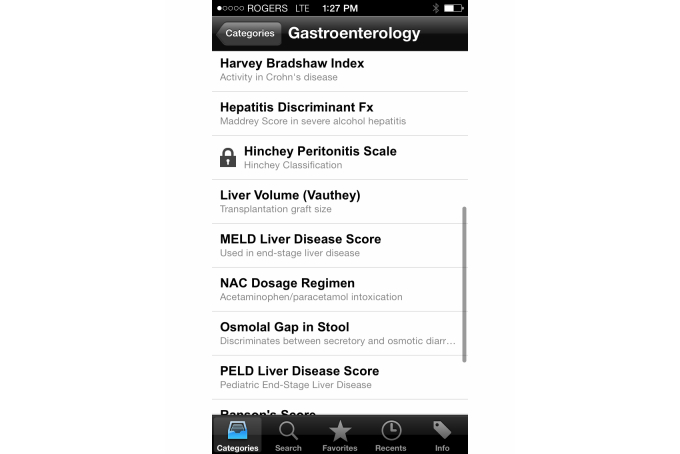
Calculations available on the CliniCalc app by Medicon Apps.

**Figure 6:**
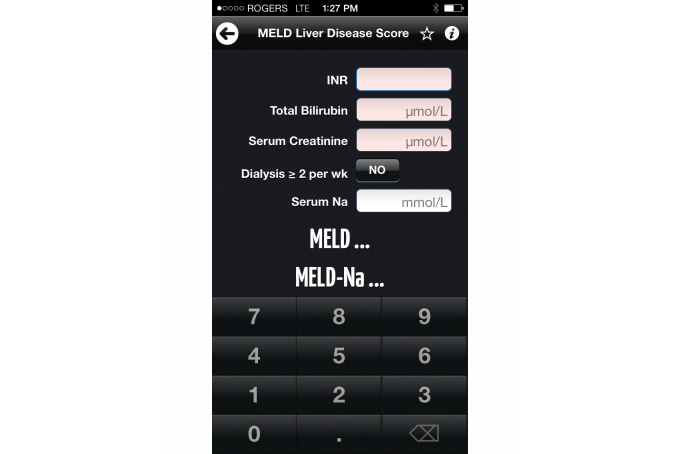
Example of a Model for End-Stage Liver Disease (MELD) Score calculation on the CliniCalc app by Medicon Apps.

Apps were tested on a single platform. Each function of each app was tested using the same 10 variations of data input, including 2 extremes and 8 middle values. The test cases were validated with clinicians for face validity. The aim of the different test cases was to produce variation in scores that would correspond to the different levels of severity that the functions contained. All the variations were recorded on an Excel spreadsheet. Answers from an app were considered correct if they were the same result as the calculation conducted using Excel with rounding error. All testing was conducted twice to reduce error. If an incorrect score was acquired, it was rechecked by another person. For each case, correct scores received a score of 1 and incorrect scores received a score of 0. Examples of calculator apps are shown in Figures 3-6.

**Table 1:** List of calculation functions.

Function	Description	# physicians choosing
CHADS_2_	Scoring system for risk of stroke in atrial fibrillation (congestive heart failure, hypertension, age 75 years or older, diabetes mellitus, previous stroke or transient ischemic attack)	5
Child-Pugh score	Classification system for severity of liver disease	5
Wells’ PE score	Scoring system for risk of pulmonary emboli (PE)	5
4T score	Scoring system for risk of heparin-induced thrombocytopenia (thrombocytopenia, timing, thrombosis, other)	4
ABCD2	Scoring system for risk of stroke after transient ischemic attack (TIA)-like symptoms (age, blood pressure, clinical features, duration of symptoms, and diabetes)	4
BMI	Body mass index	4
CIWA-Ar	Clinical Institute Withdrawal Assessment (CIWA) for Alcohol scale, revised	4
Corticosteroid conversion	Approximate equipotent dose conversions between different corticosteroids	4
HAS-BLED	Scoring system for risk of bleeding on anticoagulation (hypertension, abnormal renal/liver function, stroke, bleeding history or predisposition, labile international normalized ratio, elderly, drugs/alcohol concomitantly)	4
Creatinine clearance	Estimate of creatinine clearance by Cockcroft-Gault equation	4
MELD	Model for End-Stage Liver Disease (MELD) Scoring system for severity of liver disease, typically with United Network for Organ Sharing (UNOS) modifications	4
TIMI-STEMI	Thrombolysis in myocardial infarction (TIMI) risk stratification system after ST-elevation MI (STEMI)	4
TIMI-NSTEMI	TIMI risk stratification system after non–ST-elevation MI (NSTEMI)	4
CCS Angina Score^a^	Canadian Cardiovascular Society (CCS) Angina Score	4
GOLD classification^a^	GOLD classification of chronic obstructive pulmonary disease (COPD)	4

^a^Removed from the list of functions to test because is a classification system without any calculations.

## Results

The inclusion and exclusion criteria enabled us to come up with a list of frequently downloaded apps that were relevant to internal medicine. Fourteen (0.87%) smartphone apps were tested out of the 1603 smartphone apps found during initial research (Figure 1; Table 2).

Results of testing the 14 apps by using 10 variations for each of the 13 calculating functions are shown in Table 3. Out of the 1240 tests conducted there were 17 errors; therefore, the overall accuracy was 98.6% (17/1240).

In terms of functions, 11 of 13 functions (85%) were 100% accurate on all apps. The Child-Pugh score and the MELD score were 97% and 95% accurate, respectively. For the Child-Pugh score, there were errors in scoring for 2 apps. In all 4 errors found, the errors caused a difference in score by 1 point which did not translate to a different Child-Pugh class.

Issues occurred with the MELD score calculations on multiple apps. Eight of 14 apps produced similar incorrect scores for the cases involving creatinines >4 mg/dL (353.6 μmol/L). For 1 case, this error translated to an increased score which then gave an elevated severity (from 52.6% mortality to 71.3% mortality). This same error was found in 8 apps. These errors appeared to be because of incomplete application of United Network for Organ Sharing (UNOS) modifications of the original MELD scoring by the apps. The UNOS modification set a maximum allowable creatinine of 4 mg/dL (353.6 μmol/L).

**Table 2:** List of apps (accessed September 13, 2013).

App name	Developer(s)	Platforms available^a^	Platform tested^a^	Version tested	Cost ($US)
Calculate by QxMD [10]	QxMD	iOS, Android, BlackBerry	iOS	3.5.3	Free
CliniCalc [11]	Medicon Apps	iOS	iOS	2.1	Free
Epocrates [12]	epocrates	iOS, Android, BlackBerry	iOS	13.6	Free
MedCalc [13]	Pascal Pfiffner and Mathias Tschopp	iOS	iOS	2.7.3	$1.99
MedCalcs [14]	Beijing Kingyee Technology Co	iOS	iOS	2.6	Free
Medical Calculator [15]	Avivonet	Android	Android	1.0	$1.99
Medical Tools [16]	Irtza Sharif	Android	Android	1.2.1	Free
MediCalc [17]	ScyMed	iOS, Android	iOS	8.0	Free
MediMath [18]	Evan Schoenberg	iOS	iOS	4.3	$4.99
Mediquations [19]	Mediquations	iOS, Android	iOS	34.1	$4.99
MedScape [20]	WebMD, LLC	iOS, Android	iOS	4.2	Free
MedSolve Medical Calculator [21]	Charles Vu	iOS	iOS	1.2.2	$0.99
Skyscape Medical Resources [22]	Skyscape	iOS, Android, BlackBerry	iOS	1.18.42	Free
UpToDate [23]	UpToDate	iOS, Android, Windows	iOS*	1.3.7^b^	$563^c^

^a^iOS: iPhone/iPad/iPod operating system.

^b^The version tested was the online version on iOS platform.

^c^Requires subscription with MobileComplete and rates vary depending on role of user, country of user, and subscription term.

**Table 3:** Accuracy of medical calculating apps.

Name of app	CHADS_2_	Child-Pugh	Wells’ PE score	4T Score	ABCD2	BMI	CIWA-Ar	Corticosteroid conversion	HAS-BLED	Creatinine	MELD	TIMI-STEMI	TIMI-NSTEMI
Calculate by QxMD	100%	100%	100%	100%	100%	100%	—	100%	100%	100%	100%	100%	100%
CliniCalc	100%	100%	100%	100%	100%	100%	—	100%	100%	100%	100%	100%	100%
Epocrates	—	—	—	—	—	100%	—	100%	—	100%	80%	—	—
MedCalc	100%	100%	100%	100%	100%	100%	100%	100%	100%	100%	90%	100%	100%
MedCalcs	100%	100%	100%	—	100%	100%	—	—	100%	100%	80%	100%	100%
Medical Calculator	100%	90%	100%	—	100%	100%	—	—	—	100%	80%	100%	100%
Medical Tools	100%	100%	100%	—	—	100%	100%	100%	—	—	80%	—	100%
MediCalc	—	100%	—	—	—	100%	—	—	—	100%	100%	—	—
MediMath	100%	70%	100%	—	100%	100%	—	—	—	100%	80%	100%	100%
Mediquations	100%	100%	100%	100%	100%	100%	100%	100%	100%	100%	100%	100%	100%
MedScape	100%	—	100%	—	—	100%	—	100%	—	100%	100%	100%	100%
MedSolve Medical Calculator	100%	100%	100%	—	—	100%	—	—	—	—	80%	100%	100%
Skyscape Medical Resources	100%	100%	100%	—	—	100%	—	—	—	100%	90%	—	100%
UpToDate	100%	100%	100%	—	—	100%	100%	100%	—	—	100%	—	100%

## Discussion

The results of the study suggest that most medical calculator smartphone apps are accurate and can confidently be used in clinical settings. From an internal medicine perspective, the free apps that were 100% accurate and contained the most functions desired by internists were CliniCalc [11], Calculate by QxMD [10], and Medscape [20]. Although most of the apps provided accurate results, it is important to be cautious while using the Child-Pugh score and MELD score on certain apps, specifically.

There is a lack of evidence on the accuracy of medical calculating apps for smartphones. Information recommending medical calculating apps only provided qualitative information on the apps, without testing accuracy [24-27]. This study determines the actual accuracy of information provided by apps.

The study highlights the need for verifying medical apps before use in patient care. Although we found smartphone apps to be quite accurate, we found errors in the smartphone calculations that were clinically significant. There are efforts in the United States by the FDA to regulate medical device apps, but it is not clear if medical calculating apps are defined as medical devices in all countries [2,28]. Medical smartphone apps may be considered devices depending on the complexity of the patient information and calculation [2]. For medical apps that provide erroneous results, although downloaded from a global app store, they likely fall under legislation of the country where they are downloaded and used. The legal ramifications could be complex. Ultimately, it is likely the responsibility of the physician to determine if their calculating app is accurate. For individual physicians, testing and verifying each calculating function of each app is not reasonable. Thus, we provide physicians with clear evidence-based advice on which current apps to use.

Apps change quickly with new apps and frequent updates. We recommend that a system be put in place to verify smartphone apps that perform medical calculations to ensure they function properly. One way this can be done is by having a third party verify the accuracy of smartphone calculations. This could be conducted similar to our study but on a larger scale, with more variations and functions tested. With a list of trustworthy and validated apps, health care professionals could more confidently integrate smartphone technology into clinical settings.

There were limitations to this study. Because of the time frame of the project and the wide range of apps available, it was determined that not every function on every app could be tested. Thus, accuracy for each app may differ for other calculating functions or other test cases. Furthermore, although apps were available on multiple platforms, we only tested 1 platform for each app. Predominantly, the iOS platform was tested because most apps were available on this platform. Another limiting factor was that the focus was on apps used by general internists; therefore, results may differ for other specialties. However, this generalist approach does provide information on accuracy of smartphone medical calculation apps used by internists.

In summary, we found that most smartphone medical calculator app functions were accurate. However, some errors were noted in some functions of some apps. Given that using smartphones as medical calculators makes them a medical device, a system to verify smartphone calculation accuracy would be useful to reduce the chance of errors affecting patient care.

## Abbreviations

CCS: Canadian Cardiovascular Society
CIWA: Clinical Institute Withdrawal Assessment
COPD: chronic obstructive pulmonary disease
FDA: Food and Drug Administration
MELD: Model for End-Stage Liver Disease
NSTEMI: non-ST elevation myocardial infarction
PE: pulmonary emboli
TIMI: thrombolysis in myocardial infarction
UNOS: United Network for Organ Sharing
